# Extension in Pott's Disease

**Published:** 1902-09-13

**Authors:** 


### EXTENSION IN POTT'S DISEASE.

Speaking at Manchester on the subject of the treatment of tuberculous disease of the spinal column, Mr. John Chiene of Edinburgh recommended the recumbent position in acute cases, and in giving this advice he insisted on care being taken as to the direction in which extension is applied. In cervical cases, he said, the weight should be applied to the head, and the head of the bed should be slightly raised so that the body may act as the counter-extending force. In lumbar cases the foot of the bed should be raised and the weight be applied to the lower limbs, the body again acting as the counter-extending force; while in dorsal cases (the bed being horizontal) double extension should be employed from the head and from both lower extremities. In all cases the triple mattress should be used, so as to facilitate nursing without disturbing the patient.

In subacute and chronic cases the jacket should be used, the pelvis being the fixed point when the disease is in the lumbar and lower dorsal regions. In disease of the middle and upper dorsal and in the lower cervical regions the jacket is to be taken as the fixed point, and the jury-mast attached to the jacket must be made to support the head; while in upper cervical caries the fixed point must be taken from the shoulders (covered by a broad poroplastic collar) on which rests a Fleming's indiarubber collar, which supports the head by pressure on the mastoid process and the occipital protuberance. In all cases it must be borne in mind that pain is an indication that the child must resume the horizontal position, while an advance in the projection is a sign of healing of the carious area equivalent to the contraction of an ulcer when healing.

